# Metal Nanoparticles as Green Catalysts

**DOI:** 10.3390/ma12213602

**Published:** 2019-11-02

**Authors:** Neel Narayan, Ashokkumar Meiyazhagan, Robert Vajtai

**Affiliations:** 1 Materials Science & NanoEngineering, Rice University, Houston, TX 77005, USA; 2 Interdisciplinary Excellence Centre, Department of Applied and Environmental Chemistry, University of Szeged, Rerrich Béla tér 1, H-6720 Szeged, Hungary

**Keywords:** nanocatalysts, reuse, catalysis, biocatalyst, sustainability

## Abstract

Nanoparticles play a significant role in various fields ranging from electronics to composite materials development. Among them, metal nanoparticles have attracted much attention in recent decades due to their high surface area, selectivity, tunable morphologies, and remarkable catalytic activity. In this review, we discuss various possibilities for the synthesis of different metal nanoparticles; specifically, we address some of the green synthesis approaches. In the second part of the paper, we review the catalytic performance of the most commonly used metal nanoparticles and we explore a few roadblocks to the commercialization of the developed metal nanoparticles as efficient catalysts.

## 1. Introduction

With accelerating pollution and environmental problems, finding an appropriate solution to impede the harming of the earth has become a critical issue [1,2,3,4]. Specifically, cost-effective and environmentally friendly approaches have become significantly important. However, the revolution of materials science and nanotechnology has steeply evolved in developing multifunctional materials for future technologies on a nanoscale. Among different fields of study, the studies on the synthesis of nano-sized particles using a green chemistry approach offer an excellent platform for developing green, economical, recyclable, and sustainable materials for future applications [5,6,7].

In a typical experiment, catalysts work by lowering the activation energy, leading towards a reaction that can run to completion without the input of external energy, which is a commodity that has recently become especially crucial. Specifically, to be more efficient, the reactions should occur under conditions where lower temperatures and pressures are prevalent. This not only increases efficiency but also effectively decreases costs on a large scale. Among a wide range of catalysts, the nanocatalysts derived from nanoparticles appear as excellent alternatives to conventional nanocatalysts due to their outstanding surface area, robustness, and stability [8,9,10,11]. Nano-sized catalysts have more open active surfaces than bulk catalysts and thereby provide efficient contact between the reactant and the catalysts. It was reported that the catalytic rate increases directly with the number of catalytic sites exposed to the reaction: a catalytic site with a larger area for reactions to occur yields a faster rate of reaction and, thus, higher catalytic activity. Also, with a larger surface area comes a more significant number of reactive sites, which helps the reaction to run to completion with higher efficiency.

The other advantage is the insolubility of the catalysts in the reaction mixture, which helps easy separation and re-use of the nanocatalysts. However, despite these benefits, this area has been relatively studied due to the complexities involved in the separation of the catalysts from the final product [12,13]. The presence of these exciting features has prompted the use of catalysts in various areas of science and technology. Recently, metal-based nanoparticles have been considered very useful for different catalysis applications. In general, metal nanoparticles serve many uses, such as increasing the thermal stability of nanofluids and deriving materials with magnetic capabilities [14,15,16,17,18,19].

## 2. Metal Nanoparticles

**Figure 1:**
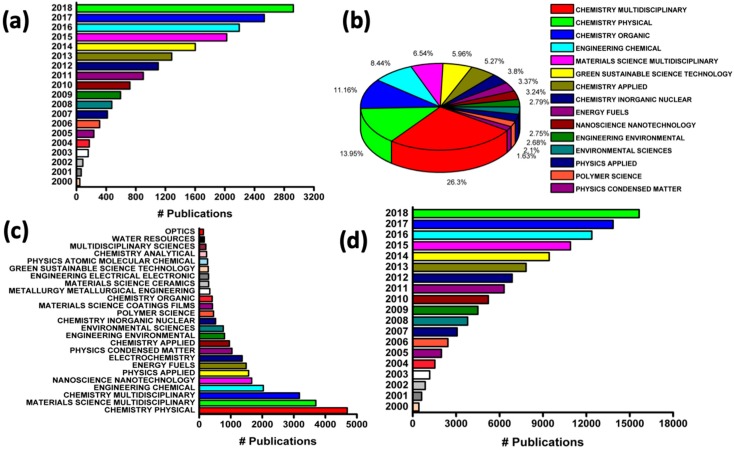
Statistics of publications between 2000 and 2018. (**a**) Approximate number of publications on green catalysts, (**b**) Approximate number of publications on metal nanoparticles, (**c**) Approximate number of publications on nanocatalysts, (**d**) Approximate percentage of publications on green catalysts. Source: Web of Science.

Metal nanoparticles are created from nanoscale matter (1–100 nm) containing both organic and inorganic materials combined with various metals [20,21,22,23]. These includes transition metals, semiconductors, alkali metals, etc. The metal part of the nanoparticles plays a great role in the use of metal nanoparticles as green catalysts due to their large surface area-to-volume ratio compared to the bulk material. This enables efficient bonding, thereby allowing reactants to bind together at metal sites, and hence, reactions occur more efficiently. Also, layered transition metal dichalcogenides (TMDs) were found to serve as rare metals in combination with nanoparticles [24]. Some examples of TMDs include molybdenum disulfide (MoS_2_), tungsten disulfide (WS_2_), molybdenum diselenide (MoSe_2_), molybdenum telluride (MoTe_2_), etc. TMDs are of particular interest specifically because of their unique properties such as their excellent optical and electrical properties and large energy density [25,26]. Figure 1 shows the importance of metal nanoparticles and green catalysts, as seen through the increase in the volume of publications in different fields of study.

Similarly, metal nanoparticles have gained substantial interest due to their interesting properties and enhanced catalytic activity. More importantly, research in this field has attracted widespread attention and is thought-provoking for the development of advanced catalysts for both fundamental studies and industrial applications. The versatility and cost-effective synthesis of metal nanoparticles have transformed these materials into the fundamental building blocks that will become more prevalent in future technologies. The primary reasons of the rapid expansion of metal nanoparticles include their easy scalability and promising catalytic activity. The presence of such exciting features, such as high surface areas and fine-tunable porosities, is considered advantageous for catalytic support [27,28,29,30].

Additionally, the electronic charges promote synergetic interactions and thus help to improve the catalytic properties significantly. The presence of these unique attributes has expanded the usage of metal nanoparticles for various applications in the optical, medicinal, technological, and biomedical areas [31,32,33,34,35,36,37]. However, surface contamination, the removal of capping agents, and particle aggregation are some of the commonly encountered problems. The most significant advantages due to the metals present in metal nanoparticles derive from their thermal and mechanical stability, electrical conductivity, strong optical and magnetic properties, and high melting and boiling points. Thermal stability plays a significant role in the use of metal nanoparticles. For example, Gould et al. reported that silver nanoparticles embedded in amorphous silicon (a-Si) exhibit higher thermal stability, thereby allowing for a more extensive range of applications [38].

## 3. Synthesis of Metal Nanoparticles

Studies on the synthesis of different nanocatalysts and their toxicities are critically important. It was reported that the toxicity and biological activity of metal nanoparticles depend on various factors such as shape, size, and surface properties [39]. Similarly, other factors, such as solubility and chemical composition, play a substantial role in the recycling process. Usually, metal nanoparticles are synthesized using different approaches depending on the desired outcome and applications. However, the synthesis procedure can be broadly classified into physical and chemical-based processes. The physical approach include flame spray pyrolysis (FSP), spray pyrolysis, electrospray process, and unconventional machining processes [40]. For example, the FSP process is particularly useful when synthesizing metal nanoparticles on a large scale [41]. In this technique, an aqueous solution consisting of metal salts is sprayed as a mist through a thin tube into a flame. Droplets form, and the metal salts turn into metal oxides. After agglomeration of the oxides, the nanoparticles begin to form and are finally collected. In spray pyrolysis, the process begins when droplets of a solution are placed in high-temperature environments. Once evaporation occurs, the droplets and the precipitated solvent are dried, precipitate annealing takes place, and the metal nanoparticles are formed. 

Aside from physical processes, various chemical processes were attempted to create metal nanoparticles. One popular method is using enzymatic biomaterials. For instance, Ahmad et al. reported a method of synthesizing cadmium sulfide (CdS) metal nanoparticles from the fungus *Fusarium oxysporum* [42]. Biomineralization, another biological process, can also occur when elements extracted from the local environment are turned into functional materials, and metal nanoparticles can thus be created [43]. The hot-injection technique has been more popularly studied recently and is considered critical due to its efficiency. Although used mainly for the growth of nanocrystals, the hot-injection synthesis procedure demonstrates the ability to generate more uniform nanocrystals, thereby suggesting ways to synthesize metal nanoparticles uniformly [44].

The Turkevich method, discovered by J. Turkevich, is one of the well-known chemical processes for the synthesis of monodispersed spherical gold nanoparticles. In general, the mechanism involves the reduction of citrate at 100 °C, followed by mixing with a gold hydrochlorate solution. Different concentrations of a citrate solution were used to achieve different particle sizes with tunable properties. The key feature of this approach is the quick nucleation process, which practically exhausts the supply of gold ions. Also, the temperature plays a critical role in controlling the particle size (i.e., at a low temperature, the nucleation process is moderate, leading to a broad size distribution) [45]. In another approach, Uppal et al. observed a gradual decrease of the diameter of gold nanoparticles due to an increase in the intensity of surface plasmon resonance [46]. The evolution followed an inverse Ostwald growth by which the size of gold nanoparticles decreases due to digestive ripening, and more mono-dispersed gold particles are formed. This technique allows for greater control over the properties of the synthesized metal nanoparticles. Besides, other techniques were reported, substituting the citrate solutions with citrate buffers to control the variability of the particle size; also, ethylenediaminetetraacetic acid was introduced to control the uniformity of the shape of the nanoparticles [47].

Apart from the above-mentioned metal nanoparticle synthesis techniques, several other green approaches have been proposed to synthesize metal nanoparticles. For instance, palladium-based nanoparticles of different morphologies were prepared at room temperature using Vitamin B1 as a source. Somorjai et al. reported the synthesis of platinum (Pt)-metalcore coated silica nanocatalyst [48]. The developed nanocatalyst was used in high-temperature catalytic reactions such as ethylene hydrogenation and CO oxidation. Among others, gold, copper, and iron-based metal nanoparticles have been rigorously studied due to their reduced toxicity and biodegradability. For a better understanding, we have tabulated various modern catalysts and their applications in Table 1.

## 4. Catalysts

The development of robust, recyclable, green catalysts is considered a significant challenge in chemistry and materials science. This area has been steadily expanding, and understanding its efficiency when using metal nanoparticles is becoming essential. More importantly, the development of green catalysts that are eco-friendly and re-usable help minimize waste disposal, and these catalytic materials are considered indispensable [78,79,80,81]. In a broader context, the catalysts have been classified as homogenous catalysts, heterogeneous catalysts, and biocatalysts. A reaction is considered homogenous when both the reactant and the catalyst are present in the same phase or physical state. These homogenous catalysts are popularly used in several industrial processes where the reactants and products are in the same (either gaseous or liquid) phase. On the other hand, heterogenous catalyst are catalysts that are in a different phase with respect to the reactants. A typical example is a solid catalyst with either liquid or gases as reactants. 

The heterogeneous catalysts are considered to be more active, efficient, and selective compared to the homogeneous catalysts. This is primarily due to the even dispersion of the metal nanoparticle catalysts in the reactants, which can provide more active sites for the reactions to occur at a faster rate. Nevertheless, the homogenous dispersibility of the catalysts makes it more challenging to separate the catalysts after completion of the reactions. Most commonly in heterogeneous catalysts, transition metals and their compounds are coated onto the surface of the catalysts to form highly active sites that lower the energy through the adsorption of reactant molecules on their surface. Heterogeneous catalysts have played a substantial role in the chemical industry for decades and they are considered essential for energy and chemical transformations [82].

## 5. Metal Nanoparticles in Catalysis Application

Metal nanoparticles play a notable role in catalysis applications. Specifically, metal nanoparticles with high surface area and more active sites promote faster reactions and increase product yield. These particles can be broadly divided into two main groups: noble-metal (Au, Pt, Ag, etc.)-supported metal nanoparticles and non-noble-metal (Fe, Cu, Ni, Co, etc.)-based nanoparticles.

In the last two decades, developments in materials science and nanotechnology have helped to achieve spectacular control over the synthesis of metallic nanoparticles with various shapes, sizes, and composition [83,84,85,86,87,88,89]. More importantly, the capability to fine-tune the structure and morphology using colloidal chemistry allows for more exceptional techniques to derive catalysts with higher active sites. These advancements act as a bridge between materials and their studies as heterogeneous catalysts. Such revolutions started when Haruta and Hutchings opened a new area of scientific debate by studying the catalytic behavior of carbon monoxide (CO) in the presence of gold nanoparticles deposited on a metal oxide [86,87,88]. They observed that sub-nanometer clusters and nanoparticles of ~1–3 nm were found to be essential for the catalysis of CO oxidation reactions and had a significant impact on the overall catalytic performance. Haruta’s group explored different procedures to optimize the particle size by controlling the calcination temperature and found that 3 nm Au nanoparticles were more efficient for the oxidation of CO. This interesting observation stimulated more studies on the synthesis of numerous metallic Au-supported nanoparticles; these nanoparticles were then widely used as catalysts for many catalytic applications [90,91,92].

Apart from gold, platinum-supported nanoparticles have been widely employed in various areas, including electrocatalytic oxidations for fuel cells, due to their high dispersibility and catalytic activity. Metallic Pt (Platinum) has been supported on different nanoparticles, including magnetic nanoparticles such as iron oxides, to form a stable and active catalyst for the oxidation of alcohols, CO, vitamin precursors, etc. [93,94,95]. Another exciting material which has been popularly studied is Palladium (Pd), to produce Pd-supported nanoparticles. Metallic Pd was supported on different substrates such as porous carbons, nanoparticles, polymers, etc. to derive active catalysts for several catalytic applications [96,97,98,99]. Overall, metal nanoparticles containing Pt, Pd, Au displayed excellent activity and selectivity towards the selective oxidation of alcohols under mild conditions. Recent studies also suggest that single-atom catalysts such as Pd-supported mesoporous alumina are efficient in the selective oxidation of allylic alcohols. In comparison, single-atom Pd supported on mesoporous alumina displayed a higher turnover frequency (TOF) than Au nanoparticles, despite its poor selectivity. However, the high cost and toxicity of noble-metal-supported catalysts emphasizes the need for alternatives for the development of readily available, non-toxic, and less expensive catalytic materials. 

Among many different options, non-noble-metal supported nanoparticles (based on Fe, Cu, Ni, etc.) have been widely studied due to their remarkable catalytic properties and large availability. Also, due to their tremendous catalytic activity, excellent stability, and environmental sustainability, Fe, Cu, and Ni have been considered as active players for heterogenous catalysis applications [100,101,102,103,104]. A single-atom doping of Co, Fe, Cu, Ni with nitrogen-doped carbon (N–C) was found to catalyze the oxidative esterification of alcohols effectively. It was reported that Cu-doped N–C displayed better performance; however, it showed reduced activity towards the oxidation of aliphatic alcohols. In another work, atomically dispersed Co was encapsulated into a metal–organic framework (Co–MOF) and tested for selective hydrogenation of nitroarenes. Co–Ni-based metal catalysts have also been studied for electrocatalytic water-splitting applications for the production of H_2_. Apart from Co-, Ni-, Cu-, and iron (Fe)-based nanoparticles have received much attention in recent days. It was reported that sub-nanometric Fe clusters performed much efficiently than Fe nanoparticles and catalyzed the hydrogenation of alkenes under mild conditions. Some other examples include the incorporation of magnetic Fe nanoparticles into graphene, Carbon Nanotubes (CNTs), activated carbons, and two-dimensional (2D) materials, which was found to influence their catalytic properties significantly. It has also been reported that iron oxide (FeO)-supported Au clusters are active in CO oxidation. However, there is still debate on the reactivity of different types of Au species. In a broader context, the synthesis of atomic thin metal clusters has a significant influence on the catalytic behaviors. Synthesized metal-supported nanoparticles have been widely employed in various industrial processes and for different applications such as water splitting and environmental remediation [105,106]. Considering this scenario, the controllable synthesis of atom-thick metal nanoclusters is still considered as a roadblock. Therefore, developing robust methodologies will be more critical for understanding and improving the performance of catalysis.

## 6. Summary and Outlook

Overall, a wide range of metal-supported nanoparticles has been synthesized in the recent past for a comprehensive study of catalytic applications. Collectively, the synthesis of green catalysts appears to be easily achievable, seems likely to overcome most of the environmental problems linked to the existing chemical processes, and may even be able to help manufacture products at high yields. However, the question of the sustainable preparation of metal nanoparticles arises (i.e., the use of environmentally friendly precursors, solvents, etc.). Interestingly, there are a few reports which suggest the use of bio-derived materials such as starch and a range of plant-derived materials for the preparation of eco-friendly metal nanoparticles containing Au, Cu, Ag, etc. These metal nanoparticles are set upon different substrates, including CNTs and proteins such as soybeans, poly-L-lysine, etc., to develop bio-inspired hybrid materials [107,108,109,110].

Another possibility is the encapsulation of metal nanoparticles on biopolymers, such as chitosan, cellulose, etc., which are sometimes used for the removal of chlorine in the water. Similarly, these nanoparticle-supported substrates can be utilized in many applications, including hydrogen sorption, environmental remediation, and fuel cell usage. Despite the possibility of the production of highly active and selective catalysts through sustainable routes, we still are far from the implementation of the existing techniques. The other primary concern is toxicity, which is yet to be revealed, associated with the synthesized nanoparticles. Metal nanoparticles, such as those based on Pd, Pt, and Rh, are poisonous, and their toxicity levels are reported to depend on various other factors, including particle size, coordinated ligands, etc. Although Fe-, Cu-, and Ni-based catalysts are found to be less toxic than Pd-, Pt-, and Rh-based catalysts, a systematic study still needs to be carried out to meticulously evaluate the cytotoxicity and biocompatibility of these nanoparticles before their bulk development and industrialization [111,112,113].

## 7. Conclusions

In this review, we discussed the various synthesis methods and designs of a wide range of metal catalysts and also addressed some of their catalytic applications. Despite the significant achievements in this area, there are still several roadblocks to be resolved, such as: (1) Establishing easy methods to synthesize bulk quantities of less toxic, uniform, robust nanoparticles with high surface areas and selectivity properties, (2) Maintaining the catalytic properties of the nanoparticles under harsh conditions, (3) Understanding the mechanisms of the catalytic process to engineer robust catalytic systems, (4) Studying the biocompatibility of the nanoparticles, taking into consideration different factors such as particle size, coordination ligands, etc., (5) Designing various strategies for bulk production. All these obstacles need to be urgently evaluated for present and future industrial applications. Though many challenges exist and various processes have not yet been methodically evaluated, these problems are expected to be addressed in the near future, leading towards the development and commercialization of efficient catalysts for many practical applications.

## Figures and Tables

**Table 1:** Various metal nanoparticles synthesized and their catalytic properties.

Metal Nanoparticles	Catalysts	Reference
Molybdenum–Bismuth Bimetallic Chalcogenide Nanoparticles	CO_2_ to Methanol	[49]
Platinum–Antimony Tin Oxide Nanoparticles	Cathode catalysis for direct methanol fuel cells via an oxygen reduction reaction (ORR)	[50]
Cobalt Oxide Nanocrystals	Cobalt Oxide Nanocrystals with CoO nanocrystals coupled with carbon nanotubes as catalysts for chlor–alkali electrolysis systems	[51]
Iron Oxide Magnetic Nanoparticles	Catalytic oxidation of phenolic and aniline chemical compounds (Fe_3_O_4_)	[52]
Zirconia Nanoparticles	Catalysts for sol–gel synthesis, aqueous precipitation, thermal decomposition, and hydrothermal synthesis	[53]
Tin Oxide Nanoparticles	Catalysts for the reduction and photodegradation of organic compounds	[54]
Silver Nanoflakes	Silver nanoflakes on molybdenum sulfide (MoS_2_) films for the catalytic oxidation of tryptophan	[55]
Tungsten Oxide Nanoparticles	Hetero-nanostructured photoelectrodes synthesized via the atomic layer decomposition of tungsten oxide (WO_3_) combined with an oxygen evolving catalyst	[56]
Cuprous Oxide Nanoparticles	Cuprous oxide nanoparticles on reduced graphene oxide (RGO) for usage as an efficient electrocatalyst in ORR	[57]
Titanium Dioxide Nanoparticles	Carbon modified titanium dioxide (TiO_2_) can be used in daylight photocatalysis	[58]
	TiO_2_ nanoparticles and photocatalytic performance measured under a medium-pressure mercury UV lamp	[59]
Iridium Oxide Nanoparticles	Ligand-free iridium oxide nanoparticles for high electrocatalytic activity	[60]
	Reusable catalyst in 1-n-butyl-3-methylimidazolium hexafluorophosphate room-temperature ionic liquid for the biphasic hydrogenation of olefins under mild reaction conditions.	[61]
Palladium Nanoparticles	Catalytic formic acid oxidation can take place through the oleylamine-mediated synthesis of palladium nanoparticles	[62]
Gold Nanoparticles	Gold nanoparticles help to create an active catalyst for the reduction of nitroarenes in an aqueous medium when placed on top of nanocrystalline magnesium oxide	[63]
	Catalytic CO oxidation can occur under the presence of gold nanoparticles	[64]
Elemental Sulfur Nanoparticles	Catalysis occurred when elemental sulfur nanoparticles were placed on chromium (VI) with a sulfide reaction	[65]
Silica Titanium Oxide Nanoparticles	Exhibit catalytic properties that can be tested for the oxidation of saturated and unsaturated hydrocarbons	[66]
Silica Vanadium Oxide Nanoparticles	Exhibit catalytic properties that can be tested for the oxidation of saturated and unsaturated hydrocarbons	
Dendrimer-Encapsulated Metal Nanoparticles	Dendrimers can be used to control the placement and other properties of metal nanoparticles for their usage as catalysts	[20]
Imidazolium Metal Nanoparticles	Metal nanoparticles immersed in imidazolium ionic liquids exhibit unique catalytic properties	[67]
Zinc Oxide Nanoparticles	Semiconducting zinc oxide nanowires made from nanoparticles can be tested for photoluminescence properties through catalytic growth	[68]
Silver Nanoparticles	Silver nanoparticles can be used as chemically stable nanoparticles with no environmentally harmful effects on microbes under anaerobic conditions	[69]
Magnesium Oxide Nanoparticles	EXAFS spectroscopy shows that magnesium oxide is a precursor of a type of mononuclear complex of gold that can catalyze ethene hydrogenation	[70]
Calcium Oxide Nanoparticles	Calcium oxide nanoparticles can be catalyzed with pyridines in an aqueous ethanol medium	[71]
Strontium-Doped Zinc Oxide Nanoparticles	Can be created with the sol–gel method, and tests showed successful photocatalytic activity of these nanoparticles when removing methylene blue (MB)	[72]
Titanium Carbide Nanoparticles	Such nanoparticles can support platinum catalysts for methanol electrooxidation in acidic mediums	[73]
Cerium Oxide Nanoparticles	These nanoparticles with their catalytic properties can be used for a variety of biomedical applications	[74]
Antimony–Vandium Oxide Catalysts	Catalysts prepared are selective for acrylonitrile formation	[75]
Metal Nanoparticles at Mesoporous N-doped Carbons and Carbon Nitrides	Metal nanoparticles at mesoporous N-doped carbons and carbon nitrides held in Mott–Schottky heterojunctions can function as efficient catalysts	[76]
Metal Nanoparticles	Catalytic properties of metal nanoparticles can be used in the synthesis of single-walled carbon nanotubes	[77]
